# A Comparison of Hyperelastic Warping of PET Images with Tagged MRI for the Analysis of Cardiac Deformation

**DOI:** 10.1155/2013/728624

**Published:** 2013-06-13

**Authors:** Alexander I. Veress, Gregory Klein, Grant T. Gullberg

**Affiliations:** ^1^Department of Mechanical Engineering, University of Washington, Seattle Washington, Stevens Way, P.O. Box 352600, Seattle, WA 98195, USA; ^2^Synarc Inc., Newark, CA 94560, USA; ^3^Lawrence Berkeley National Laboratory, Berkeley, CA 94720, USA; ^4^Department of Radiology, University of California San Francisco, San Francisco, CA 94143, USA

## Abstract

The objectives of the following research were to evaluate the utility of a deformable image registration technique known as hyperelastic warping for the measurement of local strains in the left ventricle through the analysis of clinical, gated PET image datasets. Two normal human male subjects were sequentially imaged with PET and tagged MRI imaging. Strain predictions were made for systolic contraction using warping analyses of the PET images and HARP based strain analyses of the MRI images. Coefficient of determination *R* ^2^ values were computed for the comparison of circumferential and radial strain predictions produced by each methodology. There was good correspondence between the methodologies, with *R* ^2^ values of 0.78 for the radial strains of both hearts and from an *R* ^2^ = 0.81 and *R* ^2^ = 0.83 for the circumferential strains. The strain predictions were not statistically different (*P* ≤ 0.01). A series of sensitivity results indicated that the methodology was relatively insensitive to alterations in image intensity, random image noise, and alterations in fiber structure. This study demonstrated that warping was able to provide strain predictions of systolic contraction of the LV consistent with those provided by tagged MRI Warping.

## 1. Introduction

Diagnostic imaging technologies play a vital role in reducing the morbidity and mortality associated with heart failure, cardiac ischemia, and infarction. The assessment of regional left ventricular (LV) function is currently used as a major diagnostic and prognostic indicator in patients with cardiovascular disease [1–4]. Single photon emission computed tomography (SPECT) and positron emission tomography (PET) are commonly used for evaluation of cardiovascular disease and can allow for not only evaluation of perfusion, but with gated acquisitions these nuclear images can also be used to evaluate global cardiac function measures like ejection fraction (EF) and regional function measures such as wall motion and myocardial wall thickening. Local wall motion and thickening remain the most common methods used for evaluation of LV regional wall function in the clinical setting. They are, however, indirect measures of cardiac function. Deformation in the form of wall strain represents a direct measurement of tissue elongation and contraction. These measures provide more information on the functional health of cardiac tissue than regional wall motion [5–8], allowing for earlier and more exact diagnoses to be made.

There has been growing interest in the use of deformable image registration methods for automated segmentation [9, 10] and deformation measurement of the left ventricle directly from low resolution SPECT images [11–13]. It has been suggested that these types of models can provide accurate quantitative measures of cardiac function (EF, deformation, strain) [11–13] in order to evaluate cardiac function.

Hyperelastic warping is a deformable image registration method that can determine deformations directly from the analysis of clinical medical imaging modalities such as MRI [14, 15], ultrasound [16], and microPET imaging [17]. The objective of the following study was to perform an initial comparison of strain predictions provided by hyperelastic warping of clinical PET images with strain predictions provided by the analysis of tagged MRI images from the same individuals. The point strain predictions were compared in numerous locations throughout the LV walls. Additionally, a series of studies were conducted to evaluate the sensitivity of the warping analysis to changes in image intensity level, the addition of noise to the images, and changes in the assumed underlying LV fiber structure.

## 2. Materials and Methods 

### 2.1. Hyperelastic Warping

In hyperelastic warping, the FE representation of the LV is deformed during the registration process. The forces responsible for the registration deformation are derived from the differences in image intensity of two volumetric image data sets. The first of these image data sets is the reference image, known as the template (*T*). This is the starting point in the analysis and represents the geometry upon which the FE model is based upon. The forces responsible for deforming the FE model are functions of the differences in image intensity between the template image and one or more target images (*S*). 

#### 2.1.1. Local Image Registration

A brief description of the theory underlying hyperelastic warping image registration follows. The deformation map of the registration of the FE model representation of the template image with the target image is defined as *φ*(*X*) = *x* = *X* + *u*(*X*), where *x* represents the current (deformed) coordinates corresponding to *X*, the undeformed coordinates, and *u*(*X*) is the displacement field. *F*(*X*) is the deformation gradient which is a function of this deformation map:

(1) F(X)=∂φ(X)∂X.

The image based forces responsible local registration of the discretized template image (*T*) with a target image (*S*) [18, 19] is defined by an energy term as follows:

(2) $$\begin{array}{c} U\left(X,\varphi \right)=\frac{\psi }{2}{\left(T\left(X\right)-S\left(\varphi \right)\right)}^{2}, \end{array}$$

where *ψ* is a penalty parameter that enforces alignment of the template model with the target image.

Hyperelastic warping is the process in which an energy functional is minimized. This energy functional consists of the total hyperelastic strain energy *W* from the material model and the image based term (2). The total energy takes the form:

(3) E(φ)=∫βW(X,C)dvJ−∫βU(T(X),S(φ))dvJ,

where *J* = det⁡(*F*) is the Jacobian and *C* = *F* ^*T*^ *F* is the left Cauchy-Green deformation tensor. The first term in (3) is the hyperelastic strain energy which serves to regularize the registration process. The first variation of this term gives the weak form of the momentum equations for nonlinear solid mechanics [20], while the first variation of the functional *U* in (3) gives rise to the following image-based force term:

(4) $$\begin{array}{c} DU\left(X,\varphi \right)\cdot \eta =-\psi \left[\left(T\left(X\right)-S\left(\varphi \right)\right)\frac{\partial S\left(\varphi \right)}{\partial \varphi }\cdot \eta \right]. \end{array}$$

This term drives the discretized template deformation based on the pointwise differences in image intensity (force magnitude) and the gradients (force direction). Complete details of this formulation can be found in the following work [21, 22] as well as its application to the analysis of cardiac imaging [14, 17, 23, 24].

Hyperelastic warping assumes that a hyperelastic material model defines the behavior of the material depicted in the images. In this manner, the FE model serves a dual purpose: first it is a discretized representation of the template image used in the generation of the warping forces described previously. It also represents the LV depicted in the images due to the realistic constitutive model and material property definitions defined in the LV model. For this work, the LV was defined as a transversely isotropic material [17, 24–26] in order to define the passive mechanics of the LV. Details of the passive material model and its implementation in the warping algorithm may be found in the following [21]. Transversely isotropic constitutive models are commonly used in modeling the left ventricle [27].

### 2.2. Active Contraction Constitutive Model

In order to register the LV from the end-diastolic starting point to the end-systolic state, a physiologically realistic “time varying elastance” active contraction model [28, 29] constitutive model was utilized to contract the template FE model. The total Cauchy stress tensor **T** in the fiber direction (unit vector **a**) is defined as the sum of the active stress tensor *T* ^(*a*)^  (**a** ⊗ **a**) and the passive stress tensor generated by the passive material model **T** ^(*p*)^ as follows:

(5) $$\begin{array}{c} T=T^{\left(p\right)}+T^{\left(a\right)}\left(a⊗a\right). \end{array}$$

The active fiber stress tensor *T* ^(*a*)^ is defined as

(6) $$\begin{array}{c} T^{\left(a\right)}=T_{\max}\frac{\text{C}{\text{a}}_{0}^{2}}{\text{C}{\text{a}}_{0}^{2}+E\text{C}{\text{a}}_{50}^{2}}C_{t}, \end{array}$$

where *T*_max⁡_ is the intracellular calcium concentration and *C*_*t*_ governs the shape of the activation curve [29] and is based on literature values. The length dependent calcium sensitivity *E*Ca_50_ is governed by the following equation:

(7) $$\begin{array}{c} {E\text{Ca}}_{50}=\frac{{\left({\text{Ca}}_{0}\right)}_{\max}}{\sqrt{\exp\left[B\left(l-l_{0}\right)\right]-1}}, \end{array}$$

where (Ca_0_)_max⁡_ = 4.35 *μ*M is the peak intracellular calcium concentration, *B* = 4.75 *μ*m^−1^ governs the shape of the peak isometric tension-sarcomere length relation, *l*_0_ = 1.58 *μ*m is the sarcomere length at which no active tension develops, and *l*  is the sarcomere length which is the product of the fiber stretch $\tilde{\lambda }$ and the unloaded length *l*_*r*_ = 2.04 *μ*m.

#### 2.2.1. Subject Specific Active Contraction

Rather than rely on literature values for the active contraction, a subject specific active contraction methodology was developed and applied to globally align the LV model with end-systolic image. The amount of active contraction applied to the models was governed by the amount of intensity mismatch in the images themselves. These element based image intensity differences were averaged over the entire LV mesh. This average difference in image intensity was used to define *C*_*t*_ in

(8) $$\begin{array}{c} T^{\left(a\right)}=T_{\max}\frac{\text{C}{\text{a}}_{0}^{2}}{\text{C}{\text{a}}_{0}^{2}+E\text{C}{\text{a}}_{50}^{2}}\lambda_{a}C_{t}. \end{array}$$

The average image intensity difference given in (9) was used to define the shape of the active contraction curve for all of the elements representing the LV wall. *λ*_*a*_ is a penalty parameter that enforces alignment of the template model with the target image as follows:

(9)   Ct=1numelems∑i=1numelems(T(X,φ)−S(X,φ)),

where numelems is the number of elements comprising the LV. This formulation using the average difference in image intensities was used rather than the local differences in image intensities such as is applied in warping forces, in order to produce a uniform transmural contractile stress **T** ^(*a*)^. In this manner, the warping contractile forces are controlled by the overall registration of the model to the target image producing subject specific contraction.

### 2.3. Image Data Sets

Two 3D test cases were used for this initial evaluation of hyperelastic warping for determining systolic deformations using PET images. Two, mid-twenty, male volunteers were sequentially imaged, with PET and tagged MRI. The gated PET data were acquired on a CTI/Siemens ECAT EXACT HR scanner in list mode format using ^18^F-fluorodeoxyglucose (^18^FDG). Acquisition of emission data was begun approximately 40 minutes after injection of the isotope to allow clearance from the blood pool, and acquisition continued for 20 to 60 minutes. A retrospective respiratory and cardiac double gating procedure was used to reconstruct the images into 40 msec intervals. To simplify the analysis, data from only the end-expiration portion of the respiratory cycle were used. Images were reconstructed into 256 × 256 × 47 voxel volumes using filtered backprojection. The resulting images were resampled into standard short axis orientations with dimensions 100 × 100 × 42 voxels (1.5 × 1.5 × 3.5 mm). The left ventricle was the principal feature in the resulting field of view. A 10–30 minute transmission scan was acquired prior to injection of the isotope. This scan was combined with a 60 min blank scan to correct for the effects of attenuation. A normalization file was then used to correct the emission, transmission, and blank data on a bin-by-bin basis. 

A 1.5 T Siemens scanner was used to obtain the MRI tagged data of the same patients. Spatial modulation of magnetization (SPAMM) was used to create the tags and acquire data in short-axis planes with each image slice being synchronized to the *R* wave of the EKG signal, and sequential images were acquired at 40 ms intervals. Image data were formatted into 256 × 256 pixel matrices with a 378 FOV and 10 mm slice separation. The MRI image data sets were manually coregistered to the corresponding PET images using rigid body rotations and translations of the images. Complete details of both the PET and MRI image acquisition may be found in [30].

### 2.4. Strain Analyses

#### 2.4.1. Tagged MRI Strain Analysis

The tagged MRI image data sets were analyzed using the commercially available harmonic phase (HARP) software package (Diagnosoft HARP, http://www.diagnosoft.com). Each short axis slice of the image data sets was analyzed using 2D HARP analysis. Epicardial, midwall, endocardial, circumferential, and radial strain measurements were determined for 4 regions (anterior, posterior, septal, and lateral), resulting in 12 radial and 12 circumferential strain measurements per image slice.

#### 2.4.2. Warping Strain Analysis

**Figure 1:**
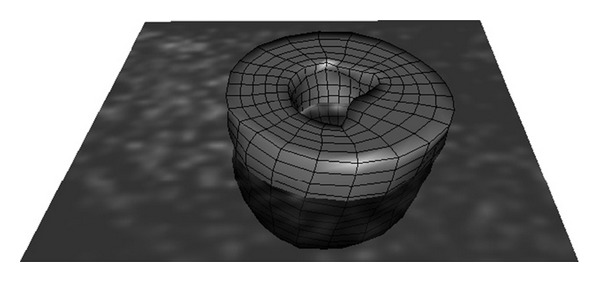
Finite element mesh of Heart 1 at the end-systolic state of the analysis superimposed on the end-systolic PET image. The FE LV model has 3366 nodes and 2712 elements.

The FE based warping models were given subject specific geometries for the two hearts using methodology described previously [17, 31]. Briefly, the 3D finite element meshes were created using surfaces based upon semiautomatic segmentation of the end-diastolic PET images (template). Each FE mesh had 3366 nodes and 2712 elements (Figure 1). The models were assigned realistic material properties [26, 32] using the material model components described previously. The fiber distributions used in the FE models were defined as −82° epicardial to 80° endocardial [33]. The end-diastolic PET image data set was used as the template image, and the end-systolic PET image was used as the target image for each case. These time points were chosen in order to match the tagged MRI data used in the HARP analyses.

The analysis was run from a time of zero to a time of 1. These times do not represent the actual time over the cardiac cycle, rather they represent the end-points of the analysis time over which the analysis of the images is made. The time allows for control of the application of the active contraction stresses (8) as well as control over the local warping forces in (4) during the analysis. For the global alignment phase, the active contraction warping was applied using a linearly increasing penalty *ψ*_*a*_ in (8) through 2/3 of the analysis (6.7 seconds) after which it was held constant until the end of the analysis. The warping penalty *ψ* in (9) and (11) was defined as linearly increasing over the course of the entire analysis [17, 31].

#### 2.4.3. Comparison of the Registration Results

Qualitative assessment of the registration between the warping epi- and endocardial surfaces and the tagged MRI image data sets was performed. The surfaces were superimposed upon both the PET and tagged MRI image data sets using the WarpLab software (http://www.mrl.sci.utah.edu/software/warplab). WarpLab is a freely distributed finite element postprocessing program that was designed to simultaneously display the FE models and the corresponding images, allowing for the visual evaluation of the deformation and registration results.

A quantitative assessment of the methodologies was made by comparing the warping radial and circumferential strain predictions with the HARP based strain measurements for the same locations. The comparisons were made using the point strains at each location for each methodology without any averaging being made. The coefficient of determination (*R* ^2^) between the two measurements was determined by correlating the strains determined by MRI with the strains predicted by warping. A Bland-Altman analysis was conducted on these strain predictions to assess the amount of agreement between the methodologies as well as to identify possible bias in the warping predictions. A regression analysis was performed on the Bland-Altman error data in order to identify trends in the warping predictions. The percent root mean squared errors (%RMSE) was used to compare the tagged MRI strain data with the strains predicted by hyperelastic warping using the tagged MRI data as the Gold Standard (10). The %RMSE was calculated as follows:

(10) %RMSE=1Nnodes∑i=1Nnodes(εtagged−εWarp)2(εtagged)2.

Here, *ε*_tagged_ represented the strain value in the tagged analysis, *ε*_warp_ was the predicted strain value for the node corresponding to this location in the warping analysis, and *N*_nodes_ was the total number of nodes in the elements representing the myocardial wall. Additionally, paired student's *t*-tests were used to assess statistical differences in the means of the strain predictions.

### 2.5. Sensitivity Studies

#### 2.5.1. Sensitivity to the Addition of Gaussian Image Noise

A series of sensitivity studies were conducted in order to evaluate the sensitivity of the strain predictions from warping to the addition of noise to the images being analyzed using methodology demonstrated in our previous studies [24, 34]. An independent additive noise model [35] was used to modify the original template and target images. The intensities of the original template and target images were considered true images *s*(*i*, *j*), where *i* and *j* represent pixel coordinates. Random noise *n*(*i*, *j*) was added to the true images to create the noisy image *I*(*i*, *j*):

(11) $$\begin{array}{c} I\left(i,j\right)=s\left(i,j\right)+n\left(i,j\right). \end{array}$$

The noise was defined by the standard deviation (*σ*_*n*_) of a zero mean normal probability distribution for noise image intensities [35], and *σ*_*i*_ was the standard deviations of the image intensities for the template and target images. The signal to noise ratio (SNR) was defined as

(12) $$\begin{array}{c} \text{SNR}=\frac{\sigma_{i}}{\sigma_{n}}. \end{array}$$

**Figure 2:**
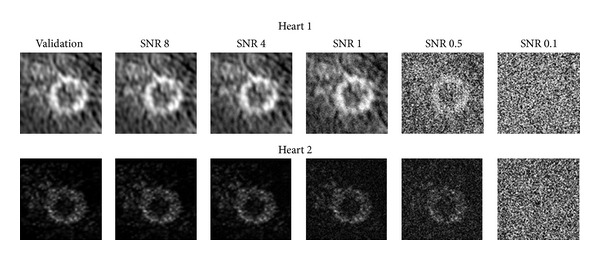
Mid ventricular slices of the image data sets used in the validation and SNR 8-SNR 0.1 analyses. The image data sets with an SNR of 0.1 demonstrate that there remains little or no image information of the LV in the images.

Images having SNR values of 8, 4, 1, 0.5, and 0.1 were evaluated (Figure 2). Coefficients of determination and %RMSE were determined for the warping analysis of these images. 

#### 2.5.2. Sensitivity to Changes in Image Intensity

**Figure 3:**
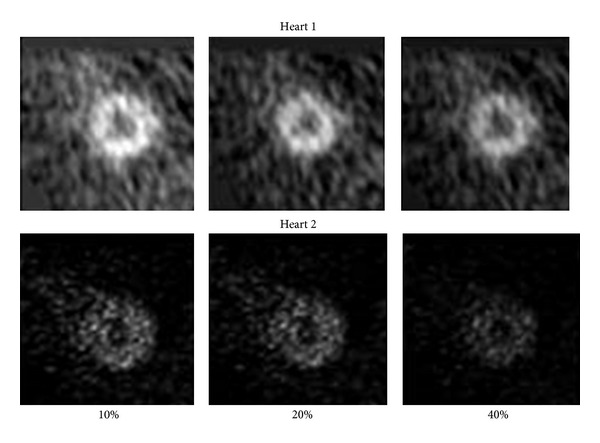
Mid ventricular slices of the images used in the image intensity sensitivity studies. These images represent the 10%, 20%, and 40% reductions in voxel intensity.

The intensities of nuclear based images represent the relative uptake of the tracer, in this case^  18^FDG, by the tissue. As the relative uptake of tracer can vary widely for nuclear imaging studies, a series of studies were carried out to determine how changes in the image intensities of the images would affect the warping strain distributions. The intensities (counts) of the template and the target image data sets were both reduced by 10%, 20%, and by 40% (Figure 3). Each case was analyzed using hyperelastic warping, and the strains were compared.

#### 2.5.3. Relative Contribution of the Active Contraction Component

The active contractile component of warping is responsible for the global alignment of the LV model with the target image. In order to determine the contribution of the active contraction component of hyperelastic warping to the overall accuracy of the strain predictions, the validation study was repeated with the active contraction component being turned off. No other warping parameters were altered. Strains were again compared with the validation study. 

#### 2.5.4. Effect of Changes in Fiber Distribution

Currently, it is extremely difficult to determine the fiber distribution of the LV for a living human subject or animal model noninvasively. Therefore, it is valuable to determine the relative sensitivity of warping to variations in the transmural fiber distribution, given that the fiber distribution used in the models will likely be based upon literature values. In order to study the effects of changes in the fiber distribution, the baseline transmural inclination angles of −82, 0, 80 (epi-, mid-, endocardial wall) used in the validation study were increased and decreased by 5%. This resulted in distributions of −86, 0, 84 and −78, 0, 76. The warping analyses were then repeated for each of these cases. Only the fiber distributions were altered with no other analysis parameters being changed. The circumferential and radial strains were compared with the tagged MRI strains as described previously.

#### 2.5.5. Effect of Fiber Distribution on the Active Contraction Component

The effect of changes in the fiber distribution on the active contraction component alone was made by evaluating the SNR 0.1 image data sets while altering the model fiber distributions. The SNR 0.1 images contain virtually no image information being completely made up of Guassian noise (Figure 2, far right panel). The differences in image intensities, in this case random noise will, on the element basis, create small image based forces. The lack of coherent image gradients means that these image based forces will be randomly oriented and will not contribute to changing the configuration of the FE LV model leaving only the active contraction to alter the LV model. Using these models, the effect of changes in the fiber distribution on the active contraction component alone was evaluated by increasing and decreasing the inclination angles for the SNR 0.1 analysis by 5% as described previously. The strain predictions from these cases were compared with the validation study and the SNR 0.1 analysis with the normal fiber distribution.

## 3. Results

### 3.1. Comparison of Registration Results

**Figure 4:**
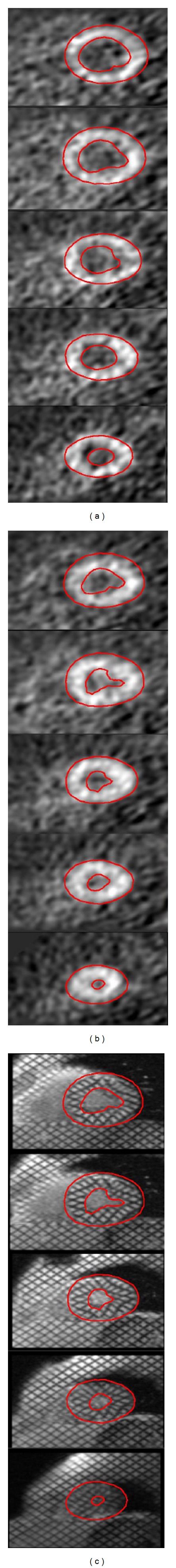
Registration comparison Heart 1. Warping analysis of Heart 1 indicates that excellent image registration was achieved on this image data set. The epi- and endocardial surfaces of the warping model correspond with those surfaces in the higher resolution MRI images on the right (c). (a) End-diastolic images (template) used in the warping analysis, (b) the end-systolic PET (target) images, and (c) the corresponding end-systolic tagged MRI images. Every fourth slice is displayed.

**Figure 5:**
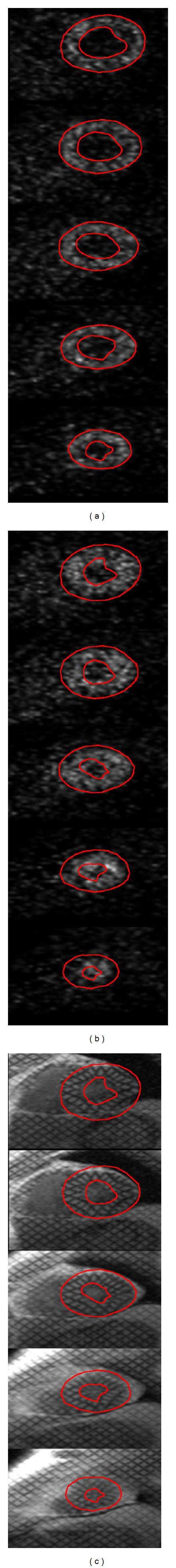
Registration comparison Heart 2. Warping analysis of Heart 2 also shows that excellent registration was achieved on this data set. (a) End-diastolic images used in the warping analysis, (b) the end-systolic PET images, and (c) the corresponding end-systolic tagged MRI images.

The visual comparison of the registered warping epi- and endocardial surfaces with the end-systolic tagged MRI images indicated that the analyses achieved excellent image registration for both hearts. The model epi- and endocardial surfaces for both models show very good correspondence with these surfaces in the tagged MRI images (Figures 4(c) and 5(c)). The FE model epi- and endocardial surfaces are displayed with the PET images used in the analysis (Figures 4(a), 4(b), 5(a), and 5(b)).

**Figure 6:**
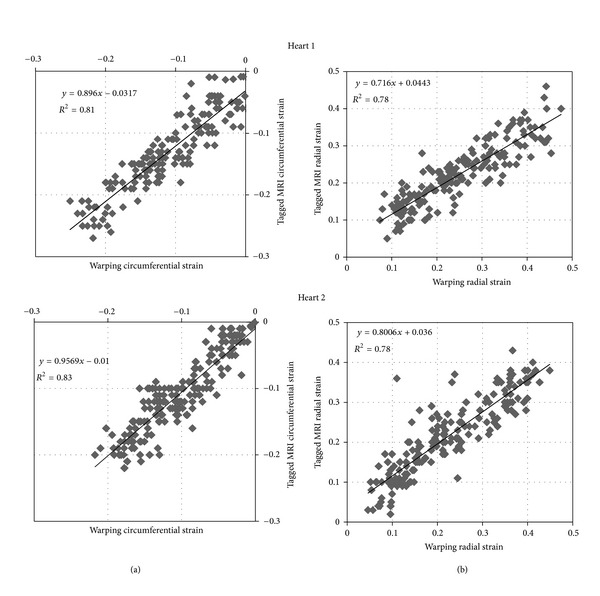
The comparisons of strains for tagged MRI analysis and the warping analysis show relatively good agreement with the comparisons of the circumferential strains (a) having *R* ^2^ values of 0.81 for Heart 1 and 0.83 for Heart 2. The comparisons of the radial strains (b) had *R* ^2^ values of 0.78 for both hearts.

**Figure 7:**
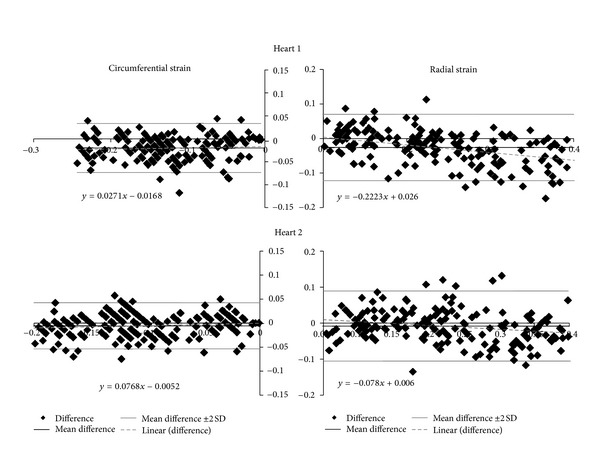
The Bland-Altman analysis comparing the warping strain results with those of the tagged MRI analysis. The warping strain predictions underestimated the strain results compared with the tagged MRI strain predictions. Warping showed little bias in the circumferential direction for both hearts with the linear regression analysis (dashed lines) showing relatively flat slopes in the circumferential strain.

The comparison of the warping strain predictions with the results of tagged MRI analysis indicated good agreement between the analysis methods (Figure 6) with *R* ^2^ values of 0.78 to 0.83. Bland-Altman analyses (Figure 7) of these results indicated that the warping strain predictions were lower than the tagged MRI analysis for both the radial and circumferential directions. In the radial direction there appears to be a tendency towards greater underestimation at higher strain values. The average strain results were not statistically different (*P* ≤ 0.01).

### 3.2. Sensitivity Studies

The results of the sensitivity studies (Tables 1 and 2) indicate that alterations in fiber distribution, moderate decreases in image intensity, and the addition of noise down to an SNR 4 for Heart 1 and SNR 1 for Heart 2 had little effect on the warping predicted strains. The results indicated that there was little change in the *R* ^2^ values and the %RMSE for these cases. The error values for the SNR 1 case of Heart 1 and SNR 0.5 for Heart 2 displayed a marked degradation of the strain predictions resulting in large increases in the error measures and decreases in the *R* ^2^ values from the addition of image noise. SNR cases below these values showed improvement in the error measures.

**Figure 8:**
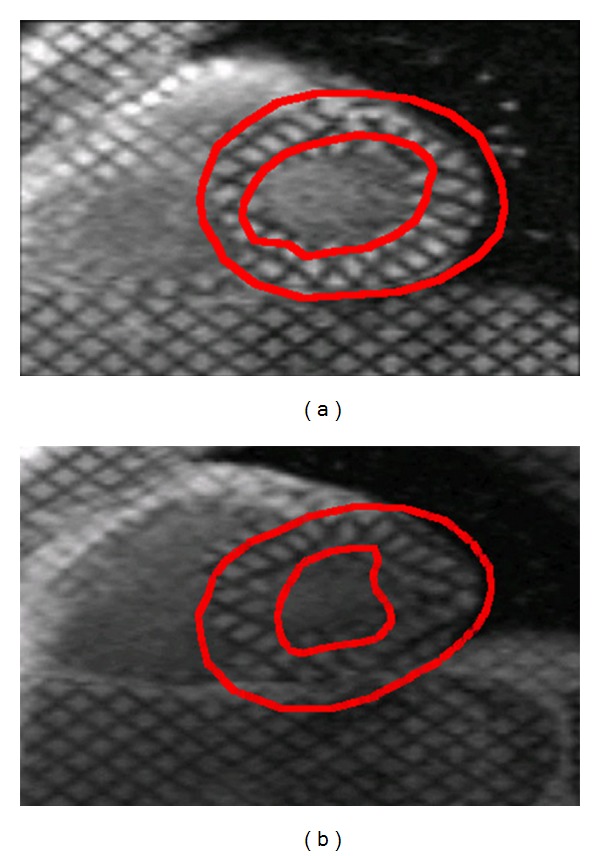
Illustrating misregistration of images when active contraction is not used. Heart 1 (a) shows anterior-laterial as well as septal misregistration for the epicardial surface. Heart 2 (b) shows posterior-lateral and septal misregistration for the epicardial surface.

The removal of the active contraction from the analysis led to regions of severe misregistration of the FE models with the end-systolic images. There were several locations of misregistration on the epicardial surfaces for each short axis slice. An example of this is given in Figure 8. The endocardial surfaces of the model simply did not register the corresponding surfaces shown in the end-systolic tagged MRI images. The effect upon the strain predictions were also pronounced with increases in the %RMSE as well as a degradation in the *R* ^2^ values for both hearts (Tables 1 and 2).

The effect of changes in the fiber distribution on the strain predictions was significant when active contraction was the only loading on the FE models. The error measures (%RMSE) increased in magnitude in the circumferential direction for Heart 1 with changes in fiber distribution. However, the %RMSE showed little change for the radial direction for Heart 1 compared with the normal fiber SNR 0.1 results (Table 3). The *R* ^2^ values for Heart 1 cases showed slight decreases for both of these strain measures. In contrast, the active contraction fiber distribution analysis for Heart 2 showed increased %RMSE for both the circumferential and radial directions compared with the normal fiber SNR 0.1 case. The *R* ^2^ values also showed large decreases in value. 

## 4. Discussion

The qualitative and quantitative evaluations of this initial validation study indicated that warping analysis of clinical PET images can provide point strain predictions consistent with those determined by tagged MRI analysis. The results show distinct trends in the comparison of the strain methodologies. The warping strain estimates showed a slight tendency towards underestimation for the circumferential direction compared with the tagged MRI analysis results as indicated by the slope of the regression lines being less than 1.0 in Figure 6. This tendency was more pronounced for the radial direction predictions where the amount of underestimation increased as indicated by the slopes of the regression lines being smaller than the values for the circumferential direction. 

The radial strain results were not surprising as there are only two to three tags across the wall (Figures 4(c) and 5(c)). Two to three tags provide a relatively low resolution for the prediction of the radial strains, and this tends to homogenize the radial strain results (Figures 6 and 7). In the warping analysis there are 10 sample points spread transmurally across the wall where the images are sampled. These are spaced approximately 0.5–1.5 mm apart depending upon where in the mesh the elements are located thus providing a substantially higher spatial resolution.

The sensitivity studies indicate that warping was relatively insensitive to changes in the fiber distribution as well as being insensitive to modest decreases in image intensities. The addition of moderate amounts of additive noise also showed little effect upon the predicted strains. However, a SNR level of 1 for Heart 1 and 0.5 for Heart 2 produced large increases in %RMSE. These errors resulted from noise induced distortions of the finite element meshes. However, as more noise was introduced (lower SNR values), the error measures improved due to the decrease and elimination of the intensity gradients in the images. These high SNR cases resulted in the magnitude of the image forces being decreased, but more importantly, the forces become randomly oriented leaving the active contraction as the primary loading on the models. These results also suggest that using a realistic material model can provide a reasonable strain distribution within regions where little image intensity information is available. The extreme example of this situation was the SNR 0.1 cases where no image information depicting the LV was left in the images.

The analysis of the SNR 0.1 image data sets allowed for the evaluation of the active contraction component without any other loading on the models. As expected, the effects of the fiber distribution were pronounced in these cases (Table 3) as all of the loading responsible for LV deformation is transmitted along the fibers. These results were in contrast to the sensitivity study where the fiber distribution was altered for the analysis of the validation image data sets. These studies indicated that alterations in fiber distribution produced little change in the error measures (Tables 1 and 2). Taken together, these results suggest that the active contraction forces only provide enough contraction to approximately align the LV model with the end-systolic image data set, and it is local warping forces that produce the deformations necessary for registration of the FE model with the target image.

The work presented in this paper is the first time point strain predictions were evaluated using clinical PET images compared with clinical tagged MRI image data sets. The results are consistent with previous warping studies. For example, warping analysis of cine-MRI images of the LV compared well with tagged MRI image analysis [36] for regional strains (average septal, lateral, posterior, and anterior) in the same patient. A comparison of warping strain predictions for the medial collateral ligament (MCL) undergoing flexion based upon the analysis of cine MRI images showed excellent agreement with strain surface marker measurements [15]. A previous, nonclinical, validation study for the use of warping with cardiac microPET imaging indicated that warping analysis strain predictions showed excellent agreement with the LV strains predicted by a forward finite element model that was used to create a set of synthetic target microPET image data sets used in the analysis [17].

*Limitations*. Obtaining deformation information from nuclear based images presents problems that are unique to these imaging modalities. PET and SPECT images are based upon the uptake in tissue of nuclear tracers imaged over relatively long periods of time (up to 60 minutes for these studies) resulting in average geometric or spatial representations of the myocardium. Patient movement, gating errors during PET acquisition and changes in heart rate during this time span could all contribute to uncompensated blurring that might affect surface representations. Furthermore, the images themselves have relatively low spatial resolutions compared with other modalities such as the tagged MRI images used for comparison in the present study. One might expect that the geometries portrayed in the images and the relatively low spatial resolution of PET would compromise the image registration. Resolution and distortions due to motion issues would then manifest in the inability of the warping methodology to produce registered models that correspond to the geometry portrayed in the higher resolution MRI images. However, the present study demonstrated that warping could produce good image registration (Figures 4 and 5) without the introduction of obvious artifacts that would have had a negative effect upon the strain predictions.

**Figure 9:**
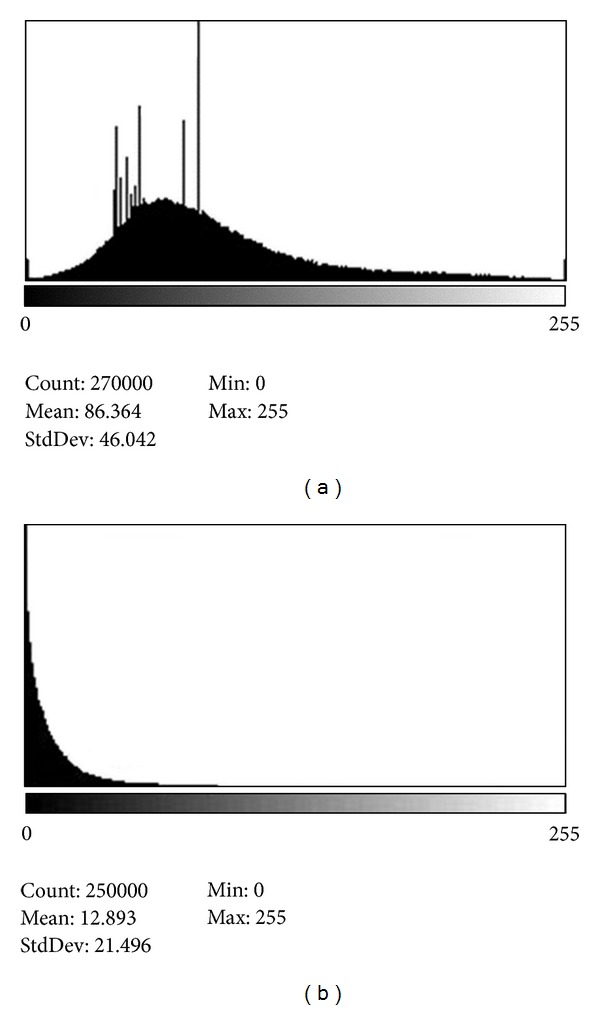
The two PET image data sets analyzed in the present study represent very different intensity distributions (full volumetric image sets). The histogram for Heart 1 (a) has a higher mean intensity value and standard deviation than those of Heart 2 (b) image data set.

**Figure 10:**
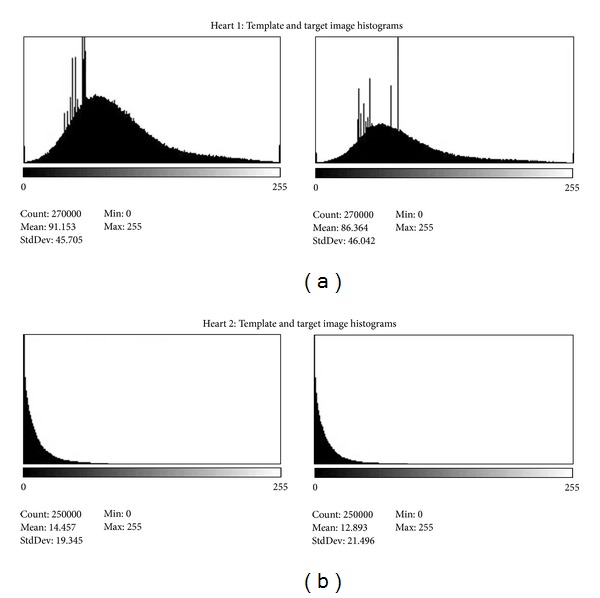
The mean image intensity of the template images in both Heart 1 (a) and Heart 2 (b) had higher mean intensity values than the final target image data sets.

Another possible source of error in the analysis of clinical PET images is that the relative uptake of tracer documented in the images will vary from patient to patient. In the present study, the histograms for the images (Figure 9) indicate that the dynamic range of the images of Heart 1 was far greater than that of Heart 2. The images of Heart 1 had a substantially higher mean intensity value as well as a greater standard deviation for the full 3D image data set than those of Heart 2. These differences in dynamic range did not appear to affect warping strain predictions compared to the tagged MRI analysis. PET based image intensity distributions may also vary over the cardiac cycle. The template image of Heart 1 had a mean intensity value of 91.2 for the histogram compared with the mean intensity histogram value in the target of 86.4 (Figure 10). This represents a 6% drop in the average intensity. In contrast, the images of Heart 2 had mean image intensity values of 14.5 in the template image and 12.9 for the target image, so an approximately 10% decrease in image intensity. The analysis results do not show any effect of the change in image intensity over the cardiac cycle. These results confirm those of our previous work [17] where intensity differences between the template and target image data sets did not affect the strain predictions. These results support the idea that systolic contraction “brightening” due to partial volume effects may have little or no effect on the strain predictions.

Warping can produce 3D Greene-Lagrange strain fields, rather than just the in-plane strain predictions as was provided by the tagged MRI analysis used in the present study. One of the primary limitations of 2D, in-plane, analysis methods is that it neglects to account for the effects of through plane motion of the heart. In a given image plane, the tissue seen and being analyzed at the reference configuration (end-diastole) is not the same tissue that is seen in this same image plane at end of the analysis (end-systole). As the base moves toward the apex, the tissue moves through the image plane. Through plane motion likely contributed to the differences in the strain predictions between the two methods. 

One of the primary limitations of the present study was that only two image data sets were available for analysis. A larger number of image data sets would have allowed for greater confidence in the results. However, even with just the two image data sets, the results suggest that the point strain predictions made from the warping analyses were consistent with those obtained using tagged MRI analysis. 

Another limitation was that the registration analyses were made on images of normal hearts. The analysis of cardiac PET images with perfusion defects from ischemia or infarction has direct clinical relevance for the use of this technology. This being the case, a possible problem with hyperelastic warping would be that the active contraction component could cause an underestimation of the strains within regions having moderate perfusion defects. This would be the case where the average intensity difference in the images (12), that drives the active contraction component of warping, would be the primary loading on the elements within the defect rather than the forces derived from the local differences in image intensity (11). This issue can be addressed in two ways. First, the active contraction penalty value used to approximately register the model with the target images was the minimum that provided reasonable alignment as subjectively judged by the user. Therefore, this methodology will likely be adequate for slight to moderate perfusion defects. Second, the analysis of images that contain one or more low perfusion regions will likely require use of additional methods in order to obtain accurate strain predictions within these segments. This would involve the identification of the elements that are within the perfusion defect(s) in order to alter the analysis parameters. Software has been developed that can be used to directly identify the elements that lie within a perfusion defect [37]. The material properties of these elements can then be altered, such that no active contraction is applied within these regions. 

The noise component added to the images for the SNR analyses did not represent the type of noise found in PET imaging. The noise found in reconstructed PET data does not have a Gaussian distribution [38]. Additionally, the additive noise model that was applied to the validation PET images was overly simplistic, as there are numerous sources for noise in PET imaging, including the noise associated with the numerous corrections that are applied (randoms, attenuation, etc.) as well as the noise associated with the image reconstruction process itself (e.g., filter back projection). Each of these sources of noise introduces its own noise distribution to the overall noise in the images. It would have been difficult in the present study to reproduce the complex processes associated with the noise generation found in PET imaging.

## 5. Conclusions

The present study has indicated that hyperelastic warping was able to provide reasonable strain predictions through the analysis of clinical PET images depicting systolic contraction that were consistent with those obtained using the HARP analysis of tagged MRI images. The sensitivity studies indicated that the methodology was relatively insensitive to moderate alterations in image intensity and image noise as well as alterations in the assumed fiber structure. Warping analysis of PET images appears to be able to provide a relatively robust method to obtain estimates of wall strains in the human LV.

## Figures and Tables

**Table 1:** Sensitivity results for Heart 1. The sensitivity study results for the Heart 1 analyses indicate that warping was relatively insensitive to image noise, moderate decreases in image intensity, and changes in fiber orientation. An SNR level of 1 (bold) caused severe degradation of the registration results with large increases in %RMSE and a reduction of the *R* ^2^ values. The lack of active contraction in the analysis also resulted in degradation of the error measures.

	Cir. strain		Radial strain	
	%RMSE	*R* ^2^	%RMSE	*R* ^2^
Validation study	0.098	0.813	0.138	0.780
SNR 8	0.109	0.813	0.122	0.780
SNR 4	0.109	0.811	0.133	0.798
SNR 1	**0.236**	**0.220**	**0.196**	**0.210**
SNR 0.5	0.280	0.770	0.439	0.790
SNR 0.1	0.131	0.786	0.145	0.750
Reduction in fiber angle (+5%)	0.105	0.813	0.143	0.796
Increase in fiber angle (−5%)	0.116	0.810	0.122	0.800
10% reduction in intensity	0.117	0.820	0.116	0.780
20% reduction in intensity	0.116	0.810	0.139	0.800
40% reduction in intensity	0.129	0.783	0.139	0.790
No active contraction	0.181	0.770	0.191	0.750

**Table 2:** Sensitivity results for Heart 2. The results of Heart 2 analyses show similar trends to those seen in the Heart 1 analyses. An SNR level of 0.5 (bold) caused severe degradation of the registration results also with large increases in %RMSE and a reduction of the *R* ^2^ values.

	Cir. strain		Radial strain	
	%RMSE	*R* ^2^	%RMSE	*R* ^2^
Validation study	0.093	0.830	0.113	0.780
SNR 8	0.095	0.824	0.113	0.773
SNR 4	0.099	0.820	0.113	0.773
SNR 1	0.099	0.821	0.113	0.804
SNR 0.5	**0.329**	**0.094**	**0.412**	**0.080**
SNR 0.1	0.114	0.790	0.124	0.770
Reduction in fiber angle (+5%)	0.092	0.830	0.110	0.780
Increase in fiber angle (−5%)	0.092	0.840	0.100	0.790
10% reduction in intensity	0.092	0.850	0.100	0.800
20% reduction in intensity	0.095	0.840	0.100	0.790
40% reduction in intensity	0.096	0.830	0.110	0.790
No active contraction	0.156	0.638	0.214	0.612

**Table 3:** Effect of changes in fiber distribution on the active contraction component alone for Hearts 1 and 2. Changes in inclination angle had a direct effect upon the strain predictions for the SNR 0.1 cases, particularly in the circumferential direction strain predictions.

	Cir. strain		Radial strain	
	%RMSE	*R* ^2^	%RMSE	*R* ^2^
Heart 1				
** **Validation study	0.098	0.813	0.138	0.780
** **SNR 0.1 normal fiber	0.131	0.786	0.145	0.750
** **SNR 0.1 + 5% IA	0.212	0.745	0.144	0.735
** **SNR 0.1 − 5% IA	0.160	0.753	0.142	0.737

Heart 2				
** **Validation study	0.093	0.830	0.113	0.780
** **SNR 0.1 normal fiber	0.114	0.790	0.124	0.770
** **SNR 0.1 + 5% IA	0.156	0.630	0.214	0.600
** **SNR 0.1 − 5% IA	0.247	0.302	0.331	0.500

IA: inclination angle.
